# Adaptability and Social Support: Examining Links With Psychological Wellbeing Among UK Students and Non-students

**DOI:** 10.3389/fpsyg.2021.636520

**Published:** 2021-02-05

**Authors:** Andrew J. Holliman, Daniel Waldeck, Bethany Jay, Summayah Murphy, Emily Atkinson, Rebecca J. Collie, Andrew Martin

**Affiliations:** ^1^Department of Psychology and Human Development, University College London, London, United Kingdom; ^2^School of Psychological, Social and Behavioural Sciences, Coventry University, Coventry, United Kingdom; ^3^Department of Social Care and Social Work, Manchester Metropolitan University, Manchester, United Kingdom; ^4^School of Education, University of New South Wales, Kensington, NSW, Australia

**Keywords:** adaptability, social support, psychological wellbeing, psychological distress, mental health, students

## Abstract

The purpose of this multi-study article was to investigate the roles of adaptability and social support in predicting a variety of psychological outcomes. Data were collected from Year 12 college students (*N* = 73; Study 1), university students (*N* = 102; Study 2), and non-studying members of the general public (*N* = 141; Study 3). Findings showed that, beyond variance attributable to social support, adaptability made a significant independent contribution to psychological wellbeing (life satisfaction, psychological wellbeing, flourishing, and general affect) and psychological distress across all studies. Beyond the effects of adaptability, social support was found to make a significant independent contribution to most wellbeing outcomes (but not psychological distress in university students). In a multi-group analysis comparing predictors of psychological wellbeing in university students and non-studying adults, where the same outcome measures were used (Study 4; *N* = 243), it was found that adaptability played a stronger role (relative to social support) for university students, whereas social support played a stronger role for non-studying adults. Finally, (contrary to expectations) there was no evidence of an interaction between adaptability and social support predicting psychological outcomes—adaptability and social support operated as independent main effects. These findings demonstrate the importance of adaptability and social support in uniquely predicting psychological wellbeing in different sample groups. It is argued here that these two factors, should be given greater consideration in discussions of psychological wellbeing, and are relevant to psychological wellbeing at different major developmental life stages.

## Introduction

There are growing concerns about mental health and wellbeing across the globe and in a range of contexts, such as in sport, the workplace, and in education: subsequently, it has become an international priority to ensure that people achieve the highest standard of mental health and wellbeing (World Health Organization, 2019). A growing literature has demonstrated the importance of a person’s adaptability (that is, cognitive, behavioral, and emotional adjustment in situations of novelty and uncertainty, Martin et al., 2012, 2013) in predicting academic (see Holliman et al., 2020) and occupational outcomes (e.g., Collie and Martin, 2017; Martin et al., 2019); however, research examining its association with psychological wellbeing is sparse. Moreover, there is a need to examine the role of adaptability in different contexts to enhance heterogeneity and generalizability (Zhou and Lin, 2016) and to examine adaptability alongside social support (a well-established predictor of psychological wellbeing, e.g., Ryan and Deci, 2017; Li et al., 2018), so their respective unique effects can be determined (e.g., Burns et al., 2018). To fill the gap, the present work examines the extent to which the resources of adaptability and social support are related to a range of psychological wellbeing outcomes among three separate samples: adolescent Year 12 college students and university students (educational contexts characterized by change, novelty, and uncertainty, Holliman et al., 2020), and non-studying adults.

### Year 12 College Education and Higher Education

In the United Kingdom, it is a legal requirement for children to stay in full-time education until the age of 16 years; however, post-16 education (e.g., Year 12 college education) and higher education is not compulsory. As such, the transition to post-16—intensified further in higher education—is markedly different from earlier education levels, not only in terms of legality, but also in terms locale (students may need to change school), social networks (previous social circles may cease and new ones may develop), subject focus and depth (students are able to choose subjects not offered in pre-16 education), and independence, autonomy-control, and responsibility (learning is less personalized with greater demands on planning and time management skills). This period also overlaps substantially with the transition through late adolescence and into adulthood which is widely recognized as a turbulent time (Burnett and Blakemore, 2009). It is clear then, that this is a period of significant change for students that, if not managed effectively, may adversely affect an individual’s healthy functioning (Hysenbegasi et al., 2005; Hudson, 2006; White and Rae, 2016).

Indeed, a connected literature among university and college students, has shown a rise in psychological distress (e.g., stress, anxiety, and depression) and a decline in psychological wellbeing (Hagell, 2012; Prince’s Trust, 2018). For example, the transition to post-16 college education has been identified as emotionally challenging for students (Hysenbegasi et al., 2005; Hudson, 2006; White and Rae, 2016) with heightened pressures reportedly affecting their mental health and life satisfaction (National Union of Teachers, 2015; Cowburn and Blow, 2017). A similar picture emerges in higher education, where the number of university students disclosing mental health conditions has increased fivefold while suicides rates have increased by 79% (Thorley, 2017); although, it is possible these findings may be explained, in part, via the increase in national initiatives encouraging discussions about mental health among young people and in the general community. It has also been reported that the wellbeing of university students is around 20% lower than the general UK population (Unite Students, 2016). It would seem important and timely then, to investigate factors that might give rise to psychological wellbeing outcomes among these student populations; however, it is equally important to understand and promote psychological wellbeing in the general population (World Health Organization, 2019).

### Conservation of Resources (COR) Model

Psychological functioning (i.e., wellbeing and distress) can be accounted for via the conservation of resource theory (Hobfoll, 1989, 2001; Hobfoll et al., 2018), which proposes that individuals procure and preserve “resources” for survival purposes. It follows that, when individuals are not facing stressful situations, they seek to develop a surplus of these resources in order to protect them against any future stress and potential loss of resources which, in turn, promotes a sense of positive psychological wellbeing (Cohen and Willis, 1985). Conversely, the model also purports that if individuals are not in a position to gain a surplus of resources, they will find themselves in a vulnerable position and they will attempt to prevent a loss of resources by engaging in self-protective behaviors (Hobfoll, 1989).

Resources then, under the COR model, are regarded as protective factors shielding individuals from stress and strain, that also influence the management of future stressful situations (Hobfoll, 2001). These have been typically classified into “personal resources” (individualistic and internal) and “conditional resources” (environmental and contextual), and it is argued that both resources are important for coping with stressful situations and encouraging a sense of positive psychological wellbeing (Cohen and Willis, 1985; Reis et al., 2015). For example, Buzzai et al. (2020) found that dispositional variables (optimism, positive/negative affectivity, and adaptive explanatory style) and contextual variables (need-supportive interpersonal behavior) were each predictive of Italian adolescent students wellbeing. It should be noted that, while the COR model has been considered mostly in relation to adversity (i.e., stress and resilience), it seems plausible that these resources might also be important for the successful navigation (management) of changing, novel, and uncertain situations.

In the present research, we have harnessed the COR theory and model, to help understand the possible precursors of psychological wellbeing and psychological distress. Specifically, in adherence with the approach adopted by Zhou and Lin (2016), we regard the aforementioned construct of “adaptability” as the personal resource and consider “social support” as a conditional resource. These constructs—elaborated on in the sections that follow—have been identified an important for the healthy functioning of individuals (e.g., Ployhart and Bliese, 2006; Martin, 2012; Chan, 2014).

#### Adaptability

As noted, adaptability—considered a personal resource under the COR model—refers to cognitive, behavioral, and emotional adjustment in situations of novelty and uncertainty (Martin et al., 2012, 2013). It is therefore defined in terms of a “tripartite” framework that involves the management, adjustment, and modification of one’s thoughts, actions, and affect, respectively. Adaptability is grounded in several other theoretical frameworks. For example, it is grounded in a self-regulation framework whereby students monitor, control, and direct their own cognitions, behaviors, and emotions, adjusting in accordance with the demands of the situation (Zimmerman, 2002). Adaptability resonates most closely with Winne and Hadwin’s (2008) fourth phase of self-regulation (adaptation), whereby students, for example, evaluate their own “performance” in order to manage and modify (where necessary) cognitions, behaviors, and emotions, to enhance future performance. Adaptability is also grounded in lifespan theory of control (Heckhausen et al., 2010) and individual functioning approaches (Buss and Cantor, 1989), whereby students make necessary modifications in order to function more positively in one’s environment. It follows then, that adaptability is of importance across the lifespan (Martin et al., 2012), and has been found to yield positive effects for different sample groups in a number of contexts (e.g., among students, teachers, teaching assistants, and others working in organizational settings, Collie and Martin, 2017; Holliman et al., 2019, 2020; Martin et al., 2019).

A developing literature has shown that adaptability is predictive of Students’ academic outcomes across education sectors (see Holliman et al., 2020), and is also a critical element of (secondary, pre-16) Students’ psychological wellbeing, as indicated by measures of self-esteem, life satisfaction, and sense of meaning and purpose (Martin et al., 2013). A common finding, which is compatible with the COR theory and model, is that adaptability may impact upon these important outcomes via behavioral engagement; specifically, students higher in adaptability are more likely to self-regulate in other situations, such as those involving task management or persistence (positive behaviors) whereas students lower in adaptability are more likely to maneuver defensively, by self-sabotaging or by disengaging (negative behaviors), in anticipation of lower self-efficacy and poorer performance (Martin et al., 2013). There remains, however, a paucity of research examining the link between adaptability and psychological wellbeing in the context of non-compulsory education (e.g., Year 12 college and higher education); although one would anticipate a positive relationship between these constructs based on prior theorizing.

#### Social Support

Social support—considered a conditional resource under the COR model—refers to the level of support that is accessible to an individual through social ties to other individuals, groups, and the larger community (Lin et al., 1979). It is generally accepted that social support impacts upon development across the lifespan (Chu et al., 2010; Parker et al., 2012). For example, a converging literature has shown that social support is associated with psychological wellbeing (Lakey and Cronin, 2008; Moak and Agrawal, 2010; Siedlecki et al., 2013) in college samples (e.g., Berndt, 1989) and in university samples (e.g., Lee et al., 2011; Yildiz and Karadaş, 2017). Moreover, and in line with COR theory, Dollete and Phillips (2004) found that social support protected university students from psychological distress and operated as a buffer against stressful circumstances.

Recent studies have also shown that adaptability is significantly associated with social support (e.g., Burns et al., 2018). It has also been theorized in the COR model, that social support may guard personal resources, such as adaptability, to help individuals strive and overcome stressful situations (Hobfoll, 2001). This has received some empirical support (see Zhou and Lin, 2016), where the relation between adaptability and psychological wellbeing (life satisfaction) was found to be moderated by social support. However, as acknowledged by Zhou and Lin (2016), there is a need to investigate the generalizability of these findings, including among more heterogeneous samples and with improved measures (e.g., a more valid measure of social support and additional measures of psychological wellbeing). Moreover, the bulk of research has demonstrated main effects of adaptability and social support and thus tests of interactions remain something of an open empirical question.

### Summary, Rationale, and Research Questions

**FIGURE 1:**
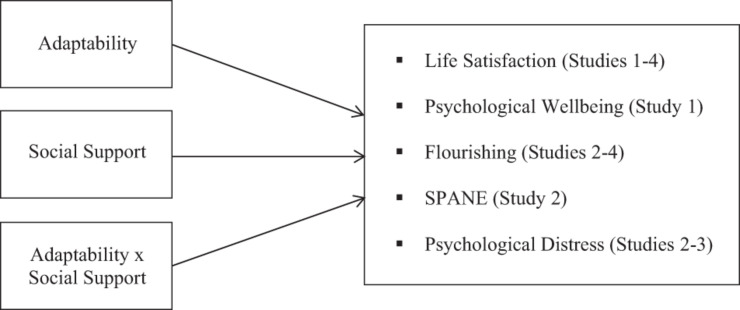
The general hypothesized model. The models control for the covariates of age and gender; SPANE, the Scale of Positive and Negative Experience.

In sum, there are growing concerns about the prevalence and impact of psychological distress (sometimes in the form of mental health conditions) and wellbeing in the general population, but particularly among students in non-compulsory education (i.e., in Year 12 college education and higher education). There is good reason to suspect that adaptability and social support—personal and conditional resources in the COR model, respectively, may be associated with psychological wellbeing outcomes in this context. However, there remains a paucity of research examining the link between adaptability and psychological wellbeing in the context of non-compulsory education. Moreover, in predicting psychological wellbeing, there is also a need to measure adaptability alongside social support given that these constructs are linked, and their respective unique associations with the outcomes can be determined (e.g., Burns et al., 2018). The recent finding that social support may moderate the observed link between adaptability and psychological wellbeing (Zhou and Lin, 2016) also merits further investigation. Moreover, it would be timely to examine the extent to which any observed patterns of association in a student sample extend to non-studying adults, who are also of importance. The current investigation extends prior work in several important ways: not only is it adding to a sparse literature in this area, but it also investigates these associations in three separate samples (addressing issues of heterogeneity and generalizability; *N* = 73 Year 12 college students, *N* = 102 university students, *N* = 141 general community adult sample). It also does so using an improved (and more holistic) measure of social support, along with a tripartite measure of adaptability, and a range of psychological wellbeing outcomes (see Figure 1, for the conceptual model underpinning this research).

Taken together, the present research addressed three major questions:

1. Do components of the COR model—assessed in this study via adaptability (personal resource) and social support (conditional resource)—contribute significantly, and independently, to psychological wellbeing outcomes among students and non-students?
2. Is there an interaction effect between adaptability and social support on psychological wellbeing outcomes among students and non-students; specifically, a moderating role of social support?
3. To what extent do adaptability or social support play similar or different roles in predicting psychological wellbeing outcomes across the two adult samples?

## Study 1

### Materials and Methods

#### Participants and Procedure

All participants in this study (*N* = 73) were recruited from a single college in the West Midlands, United Kingdom. Students were 70 percent female (*n* = 51), aged between 16 and 17 years (*M* = 16.79; *SD* = 0.41), and were currently in Year 12 enrolled on a range of Advanced Level (A Level) courses. The selection criteria for participation were not limited to any particular demographic or ability group; all students were invited to take part in this research. Participant information sheets and consent forms were distributed to all eligible participants in the Spring term of 2019. This took place during a non-academic, neutral ground seminar session to maintain student autonomy and minimize learning interference. These forms clarified the aims and nature of the research and made students fully aware of their rights prior to participation. This research, and that reported in Studies 2 and 3, received ethical approval from the Faculty Research Ethics Committee (REC) at a Higher Education Institution in the West Midlands, United Kingdom, and adheres with the British Psychological Society’s Code of Ethics and Conduct. Students willing to take part were asked to complete a paper questionnaire, which included demographic information (age, gender) along with measurement scales to assess Students’ adaptability, social support, psychological wellbeing, and life satisfaction.

#### Measures

All measures in this study were chosen on the bases that their scores have demonstrated acceptable reliability and validity among adolescent samples in other studies (e.g., Adaptability: Martin et al., 2012, 2013; Social Support: Canty-Mitchell and Zimet, 2000; Life Satisfaction and Psychological Wellbeing: Public Health England, 2015).

##### Adaptability

The Adaptability Scale (Martin et al., 2013) is a 9-item measure designed to assess Students’ cognitive, behavioral, and emotional adaptability. Participants responded to items (e.g., “I am able to adjust my thinking or expectations to assist me in a new situation”) using a 7-point Likert scale from 1 (strongly disagree) to 7 (strongly agree) (α = 0.80 in the present study).

##### Social Support

The Multidimensional Scale of Perceived Social Support (Zimet et al., 1988) is a 12-item measure designed to assess Students’ perceived level of social support relating to family, friends, and significant others. Participants responded to items (e.g., “I would describe my satisfaction with my life as …”) using a 7-point Likert scale from 1 (terrible) to 7 (delighted) (α = 0.92 in the present study).

##### Life Satisfaction

The Brief Multidimensional Students’ Life Satisfaction Scale, BMSLSS (Huebner et al., 2006) is a 6-item measure designed to assess adolescents’ satisfaction with different aspects of lives, including family, school, own self, living environment in addition to overall life satisfaction. Item three of the scale was altered from “I would describe my satisfaction with my school experience as …” to “I would describe my satisfaction with my college experience as …” to suit the context of the current study. Participants responded to items (e.g., “I would describe my life satisfaction as …”) using a 7-point Likert scale from 1 (terrible) to 7 (delighted) (α = 0.77 in the present study).

##### Psychological Wellbeing

The ONS Personal Wellbeing Domain for Young People aged 16–24 (Official National Statistics, 2014) is a 4-item measure to assess students’ general level of psychological wellbeing. Participants responded to items (e.g., “Overall how anxious did you feel yesterday”) using a 10-point Likert scale from 1 (not at all) to 10 (completely) (α = 0.71 in the present study).

##### Covariates

Gender was coded 0 or 1 for males/females. Age was a continuous variable.

#### Data Analysis

Descriptive statistics were calculated, along with reliability estimates using Cronbach’s alpha and bivariate correlations. For analyses, we used M*plus* 8.4 (Muthén and Muthén, 2017) with robust maximum likelihood (MLR) as the estimator. Substantive variables were entered into modeling as mean scores. Path analysis was conducted to examine the unique associations of adaptability, perceived social support, and their interaction with the outcome variables of life satisfaction and psychological wellbeing. Two covariates (gender and age) served as controls for all substantive variables. There were no missing data for Study 1. To calculate interaction terms, the scores of the key predictor variables were first mean-centered and an interaction term was computed by multiplying the centered predictors (Aiken and West, 1991). Because the model was fully forward, the model was fully saturated. Nonetheless, we report the comparative fit index (CFI), Tucker Lewis index (TLI), and the root-mean-square error of approximation (RMSEA). CFI and TLI values of ≥ 0.90 and ≥ 0.95 indicate adequate and good fit, respectively (Hu and Bentler, 1999). RMSEA values of ≤ 0.08 and ≤ 0.05 or less indicate adequate and good fit, respectively (Hu and Bentler, 1999).

### Results

#### Descriptive Statistics

Means, standard deviations, and bivariate correlations among the key variables are presented in Table 1. As indicated, adaptability and perceived social support were significantly positively associated with all the key variables.

**TABLE 1:** Means, standard deviations, and correlations between Study 1 variables (*N* = 73).

Variable	1	2	3	4
1. Adaptability				
2. Social support	0.24*			
3. Life satisfaction	0.43**	0.55**		
4. Psychological wellbeing	0.49**	0.42**	0.72**	
Mean	5.23	5.90	5.14	6.86
SD	0.68	0.98	0.85	1.55

*p < 0.05; **p < 0.001. Adaptability, Social Support, and Life Satisfaction were scored from 1 to 7, and Psychological Wellbeing was scored from 1 to 10, with higher scores corresponding to higher levels of each respective construct.

#### Moderated Path Analyses

**FIGURE 2:**
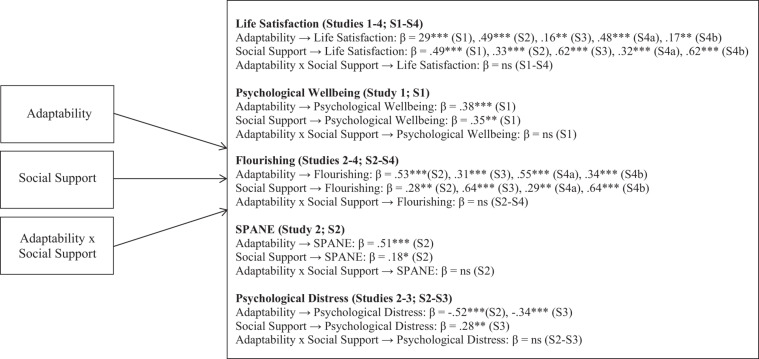
Summary of significant substantive findings, Studies 1–4. The models control for the covariates of age and gender; SPANE, The Scale of Positive and Negative Experience; S4a, Study 4 student sample, S4b, Study 4 non-student sample; ns, not statistically significant at *p* < 0.05.

The model was fully forward and so model fit was saturated: χ^2^(0) = 0, *p* = 0, CFI = 1.00, TLI = 1.00, RMSEA = 0. As Table 2 and Figure 2 shows, adaptability [β = 0.29, *p* < 0.001, 95% *CI* = (0.13, 0.45)] and perceived social support [β = 0.49, *p* < 0.001, 95% *CI* = (0.28, 0.70)] had significant positive associations with the outcome variable of life satisfaction. However, there was no interaction effect observed [β = 0.11, *p* = 0.35, 95% *CI* = (−0.33, 0.12)]. The variance explained by the predictors was 47%. Similarly, adaptability [β = 0.38, *p* < 0.001, 95% *CI* = (0.17, 0.59)] and perceived social support [β = 0.35, *p* < 0.01, 95% *CI* = (0.12, 0.59)] also had significant positive associations with the outcome variable of psychological wellbeing. Once again, there was no interaction effect observed [β = −0.10, *p* = 0.22, 95% *CI* = (−0.27, 0.06)]. The variance explained by the predictors was 42%.

**TABLE 2:** Standardized beta estimates from path analysis for Studies 1–3.

				Wellbeing outcomes				
	Adaptability	Social support (SS)	Adaptability × SS	Life satisfaction	PWBS	Flourishing	SPANE	Psychological distress
Study 1 (*N* = 73)								
Gender	−0.11	0.16	−0.03	−0.22**	−0.27**	—	—	—
Age	0.03	−0.09	0.08	−0.15	−0.09	—	—	—
Adaptability				0.29***	0.38***	—	—	—
Social support (SS)				0.49***	0.35**	—	—	—
Adaptability × SS				0.11	−0.10	—	—	—
*R*^2^	0.02	0.04	0.01	0.47	0.42	—	—	—
Study 2 (*N* = 102)								
Gender	−0.16*	0.17	0.08	0.09	—	0.08	−0.07	0.07
Age	0.16	0.14*	0.10	−0.14	—	0.02	−0.15	0.21*
Adaptability				0.49***	—	0.53***	0.51***	−0.52***
Social support (SS)				0.33***	—	0.28**	0.18*	−0.11
Adaptability × SS				0.05	—	−0.07	0.08	−0.09
*R*^2^	0.04	0.06	0.02	0.43	—	0.50	0.33	0.33
Study 3 (*N* = 141)								
Gender	0.04	0.07	−0.03	-0.13*	—	−0.04	—	0.14*
Age	0.22*	−0.21**	−0.05	0.05	—	0.15*	—	−0.29***
Adaptability				0.16**	—	0.31***	—	−0.34***
Social support (SS)				0.62***	—	0.64***	—	−0.28**
Adaptability × SS				−0.01	—	-0.07	—	0.09
*R*^2^	0.05	0.05	0.01	0.44	—	0.60	—	0.30

*p < 0.05; **p < 0.01; ***p < 0.001. SPANE, The Scale of Positive and Negative Experience; PWBS, Psychological Wellbeing Scale.

## Study 2

### Materials and Methods

#### Participants and Procedure

All participants in this study (*N* = 102) were recruited from a single higher education institution (university) in the West Midlands, United Kingdom. Students were 76% female (*n* = 77), aged between 19 and 38 years (*M* = 20.84; *SD* = 3.19), and were currently in Year 2 (Level 5) enrolled in either BSc (Hons) Psychology (78.4%), BSc (Hons) Criminal Psychology (12.7%), or BSc (Hons) Sport Psychology (8.8%) courses. As with Study 1, the selection criteria for participation were not limited to any particular demographic or ability group; all students in the Schools of Psychological, Social, and Behavioral Sciences, were invited to take part in this research. Participant information sheets and consent forms were distributed to eligible students in each respective program during a mandatory lecture in the Spring term of 2019. As in Study 1, these forms clarified the aims and nature of the research and made students fully aware of their rights. Students who were willing to take part then completed a paper questionnaire, which included demographic information along with measurement scales to assess Students’ adaptability, social support, life satisfaction, psychological wellbeing, positive and negative experience, and psychological distress.

#### Measures

As in Study 1, the Adaptability Scale (α = 0.86 for Study 2) and the Multidimensional Scale of Perceived Social Support (α = 0.94) were used in this study. Some alternative, additional psychological wellbeing measures were included (below), again chosen because their scores have shown acceptable reliability and validity among adult samples in other studies (e.g., Adaptability: Collie et al., 2017; Social Support: Clara et al., 2003; Life Satisfaction: Pavot et al., 1991; Flourishing: Diener et al., 2010; Positive and Negative Experience: Telef, 2015; Psychological Distress: Zenebe et al., 2021).

##### Life Satisfaction

The Satisfaction with Life Scale, SWLS (Diener et al., 1985) is a 5-item measure designed to assess Students’ general level of satisfaction with their lives. Participants responded to items (e.g., “I am satisfied with my life”) using a 7-point Likert scale from 1 (strongly disagree) to 7 (strongly agree) (α = 0.81).

##### Flourishing

The Flourishing Scale (Diener et al., 2009) is an 8-item measure designed to assess Students’ self-perceived success and psychological wellbeing. Participants responded to items (e.g., “I am optimistic about my future”) using a 7-point Likert scale from 1 (strongly disagree) to 7 (strongly agree) (α = 0.87).

##### Positive and Negative Experience

The Scale of Positive and Negative Experience (SPANE) (Diener et al., 2009) is a 12-item measure designed to assess Students’ positive and negative emotions over the last 4 weeks. Participants responded to items [e.g., “Pleasant (positive)” and “Sad (negative)”] using a 5-point Likert scale from 1 (very rarely or never) to 5 (very often or always). The negative total score (α = 0.79) is deducted from the positive total (α = 0.77) score to create a “SPANE Balance” score.

##### Psychological Distress

The Kessler Psychological Distress Scale (K10) (Kessler et al., 2002) is a 10-item measure designed to assess Students’ general level of psychological distress. Participants responded to items (e.g., “During the last 30 days, about how often did you feel hopeless?”) using a 5-point Likert scale from 1 (none of the time) to 5 (all of the time) (α = 0.71 in the present study).

##### Covariates

Gender was coded 0 or 1 for males/females. Age was a continuous variable.

#### Data Analysis

Descriptive statistics were calculated, along with reliability estimates using Cronbach’s alpha and bivariate correlations. The analyses that were conducted followed the same procedure as in Study 1. We used M*plus* 8.4 (Muthén and Muthén, 2017) with the MLR estimator. Substantive variables were entered into modeling as mean scores. Path analysis was conducted to examine the unique associations of adaptability, perceived social support, and their interaction with the outcome variables of life satisfaction, flourishing, positive and negative experience, and psychological distress. Two covariates (gender and age) served as controls for all substantive variables. Missing data (<1% for Study 2) were handled with full information maximum likelihood (FIML; Enders and Bandalos, 2001). To calculate interaction terms, the scores of the key predictor variables were first mean-centered and an interaction term was computed by multiplying the centered predictors (Aiken and West, 1991). Because the model was fully forward, the model was fully saturated. Nonetheless, we report the CFI, FLI, and RMSEA.

### Results

#### Descriptive Statistics

Means, standard deviations, and bivariate correlations among the key variables are presented in Table 3. As indicated, adaptability and perceived social support were significantly positively associated with all the psychological wellbeing variables and were significantly negatively associated with psychological distress.

**TABLE 3:** Means, standard deviations, and correlations between study 2 variables (*N* = 102).

Variable	1	2	3	4	5	6
1. Adaptability						
2. Social support	0.33**					
3. Life satisfaction	0.55**	0.47**				
4. Flourishing	0.64**	0.49**	0.65**			
5. SPANE	0.56**	0.30*	0.61**	0.67**		
6. Psychological distress	−0.52**	−0.21*	−0.50**	−0.53**	−0.71**	
Mean	5.18	5.58	4.72	5.68	1.13	2.42
SD	0.80	1.24	1.11	0.75	1.13	0.78

*p < 0.05; **p < 0.001. Adaptability, Social Support, Life Satisfaction, and Flourishing were scored from 1 to 7, and Psychological Distress was scored from 1 to 5, with higher scores corresponding to higher levels of each respective construct. SPANE, The Scale of Positive and Negative Experience. SPANE is a balance score where zero is neutral and where higher positive or negative scores correspond with stronger positive or negative emotions, respectively.

#### Moderated Path Analyses

The model was fully forward and so model fit was saturated: χ^2^(0) = 0, *p* = 0, CFI = 1.00, TLI = 1.00, RMSEA = 0. As Table 2 and Figure 2 shows, adaptability [β = 0.49, *p* < 0.001, 95% *CI* = (0.34, 0.63)] and perceived social support [β = 0.33, *p* < 0.001, 95% *CI* = (0.20, 0.45)] had significant positive associations with the outcome variable of life satisfaction. However, there was no interaction effect observed [β = 0.05, *p* = 0.53, 95% *CI* = (−0.11, 0.21)]. The variance explained by the predictors was 43%. Similarly, adaptability [β = 0.53, *p* < 0.001, 95% *CI* = (0.33, 0.74)] and perceived social support [β = 0.28, *p* < 0.01, 95% *CI* = (0.12, 0.44)] had significant positive associations with the outcome variable of flourishing (psychological wellbeing). Once again, there was no interaction effect observed [β = −0.07, *p* = 0.42, 95% *CI* = (−0.25, 0.10)]. The variance explained by the predictors was 50%. Moreover, adaptability [β = 0.51, *p* < 0.001, 95% *CI* = (0.34, 0.68)] and perceived social support [β = 0.18, *p* < 0.05, 95% *CI* = (0.02, 0.33)] had significant positive associations with the outcome variable of general affect (SPANE). However, there was no interaction effect observed [β = 0.08, *p* = 0.35, 95% *CI* = (−0.09, 0.25)]. The variance explained by the predictors was 33%. In contrast, there was a significant negative relationship between adaptability [β = −0.52, *p* < 0.001, 95% *CI* = (−0.69, −0.35)] and the outcome variable of psychological distress. However, there was no effect observed for social support [β = −0.11, *p* = 0.25, 95% *CI* = (−0.29, 0.08)], and no interaction effect observed [β = −0.09, *p* = 0.23, 95% *CI* = (−0.23, 0.06)]. The variance explained by the predictors was 33%.

## Study 3

### Materials and Methods

#### Participants and Procedure

The Study 3 sample consisted of 141 general community (non-studying) adult participants and comprised of 94 females (66.7%). This sample was drawn from online recruitment portals (e.g., www.findparticipants.com, https://www.reddit.com/r/SampleSize/) and from an original sampling frame of *N* = 233. However, 92 of the original sampling frame declared they were an *active* student and were thus removed from the dataset, as Study 3 was interested in a non-studying post-school sample. The selection criteria for participation were limited to participants aged 18+. Participants were aged between 18 and 71 years (*M* = 36.67; *SD* = 14.00). Most of the sample were of American (53.9%) or British (11.3%) nationality. Further, most of the sample identified as white/Caucasian (77.3%). As in Studies 1 and 2, all ethical obligations were adhered to (i.e., informed consent, anonymity, right to withdraw etc.).

#### Measures

As in Study 2, the Adaptability Scale (α = 0.92 for Study 3), the Multidimensional Scale of Perceived Social Support (α = 0.93), the Satisfaction with Life Scale, SWLS (α = 0.89), and the Flourishing Scale (α = 0.91) were used for this study. An additional psychological distress measure was also included (below), again chosen based on its scores demonstrating acceptable sample reliability and validity as evidenced in prior work (e.g., Osman et al., 2012).

##### Psychological Distress

The Depression, Anxiety, and Stress Scales (DASS-21) (Lovibond and Lovibond, 1995; Henry and Crawford, 2005) is a 21-item measure designed to assess participants’ general level of psychological distress. Participants responded to items (e.g., “I found it hard to relax”) using a 4-point Likert scale from 0 (did not apply to me at all) to 3 (applied to me very much, or most of the time) (α = 0.90).

##### Covariate

Gender was coded 0 or 1 for males/females. Age was a continuous variable.

#### Data Analysis

Descriptive statistics were calculated, along with reliability estimates using Cronbach’s alpha and bivariate correlations. The analyses that were conducted followed the same procedure as in Study 1. We used M*plus* 8.4 (Muthén and Muthén, 2017) with the MLR estimator. Substantive variables were entered into modeling as mean scores. Path analysis was conducted to examine the unique associations of adaptability, perceived social support, and their interaction with the outcome variables of life satisfaction, flourishing, and psychological distress. Two covariates (gender and age) served as controls for all substantive variables. Missing data (<1% for Study 3) were handled with FIML. To calculate interaction terms, the scores of the key predictor variables were first mean-centered and an interaction term was computed by multiplying the centered predictors (Aiken and West, 1991). Because the model was fully forward, the model was fully saturated. Nonetheless, we report the CFI, FLI, and RMSEA.

### Results

#### Descriptive Statistics

Means, standard deviations, and bivariate correlations among the key variables are presented in Table 4. As indicated, adaptability and perceived social support were significantly positively associated with all the psychological wellbeing variables and were significantly negatively associated with psychological distress.

**TABLE 4:** Means, standard deviations, and correlations between study 3 variables (*N* = 141).

Variable	1	2	3	4	5
1. Adaptability					
2. Social support	0.16				
3. Life satisfaction	0.27**	0.63**			
4. Flourishing	0.47**	0.66**	0.73**		
5. Psychological distress	−0.41**	−0.27**	−0.48	−0.60	
Mean	5.40	4.80	4.11	4.98	0.84
SD	1.00	1.48	1.45	1.25	0.64

*p < 0.05; **p < 0.001. Adaptability, Social Support, Life Satisfaction, and Flourishing were scored from 1 to 7, and Psychological Distress was scored from 0 to 3, with higher scores corresponding to higher levels of each respective construct.

#### Moderated Path Analyses

The model was fully forward and so model fit was saturated: χ^2^(0) = 0, *p* = 0, CFI = 1.00, TLI = 1.00, RMSEA = 0. As Table 2 and Figure 2 shows, adaptability [β = 0.16, *p* < 0.01, 95% *CI* = (0.05, 0.28)] and perceived social support [β = 0.62, *p* < 0.001, 95% *CI* = (0.46, 0.77)] had significant positive associations with the outcome variable of life satisfaction. However, there was no interaction effect observed [β = −0.01, *p* = 0.84, 95% *CI* = (−0.12, 0.10)]. The variance explained by the predictors was 44%. Similarly, adaptability [β = 0.31, *p* < 0.001, 95% *CI* = (0.17, 0.46)] and perceived social support [β = 0.64, *p* < 0.001, 95% *CI* = (0.52, 0.76)] had significant positive associations with the outcome variable of flourishing (psychological wellbeing). Once again, there was no interaction effect observed [β = −0.07, *p* = 0.30, 95% *CI* = (−0.21, 0.07)]. The variance explained by the predictors was 60%. Moreover, adaptability [β = −0.34, *p* < 0.001, 95% *CI* = (−0.49, −0.19)] and perceived social support [β = 0.28, *p* < 0.01, 95% *CI* = (−0.43, −0.12)] had significant negative associations with the outcome variable of psychological distress. However, there was no interaction effect observed [β = 0.09, *p* = 0.28, 95% *CI* = (−0.25, 0.07)]. The variance explained by the predictors was 30%.

## Study 4

As is evident, Studies 2 and 3 not only include the same predictor variables (adaptability, social support, and covariates), they also include two common psychological outcome variables: life satisfaction and flourishing. This commonality enables formal multi-group comparisons to ascertain if adaptability and social support have similar or different effects on psychological outcomes across these two adult samples.

### Materials and Methods

#### Participants and Procedure

Study 4 therefore combined the samples from Studies 2 and 3 above to compare the roles of adaptability and social support in relation to life satisfaction and flourishing, and to ascertain whether there were any differences in these associations across the university (*N* = 102) and non-studying adult samples (*N* = 141).

#### Results

Once again using M*plus* 8.4 (Muthén and Muthén, 2017) with the MLR estimator and controlling for gender and age, path analysis results are displayed in Table 5 and Figure 2 (because the interaction effect was not significant in the previous studies, it was not included here). Understandably, results are very similar to those shown in Studies 2 and 3 above. Notably, however, we were able to compare the strength of the associations among the substantive variables using Wald tests of difference. These tests revealed three significantly different paths across the two samples. In the university sample, the path from adaptability to life satisfaction was significantly stronger than the same path in the adult sample [Wald(1) = 10.20, *p* = 0.001]. In contrast, the paths from social support to life satisfaction [Wald(1) = 7.34, *p* = 0.007] and flourishing [Wald(1) = 9.78, *p* = 0.002] were significantly stronger for the adult sample. Thus, adaptability appeared to be more central for the university sample, whereas social support was more central for the adult sample.

**TABLE 5:** Summary of path analysis for variables predicting wellbeing outcomes for Study 4.

Variable	Adaptability	Social support	Life satisfaction	Flourishing
**University sample (*N* = 102)**				
Gender	−0.16*	0.17	0.10	0.07
Age	0.16	0.14*	−0.14	0.01
Adaptability			0.48***	0.55***
Social support (SS)			0.32***	0.29**
*R*^2^	0.04	0.06	0.42	0.49
**Non-student sample (*N* = 141)**				
Gender	0.04	0.07	−0.13*	−0.04
Age	0.22*	−0.21**	0.05	0.15*
Adaptability			0.17**	0.34***
Social support (SS)			0.62***	0.64***
*R*^2^	0.05	0.05	0.44	0.60

*p < 0.05; **p < 0.01; ***p < 0.001.

## Discussion

### Findings of Note

In the current set of studies, we examined the roles of adaptability and social support in a range of psychological wellbeing outcomes across three different samples. We found that in each case, adaptability and social support had a significant independent association with life satisfaction, flourishing, and personal wellbeing. This finding was consistent with conservation of resource theory (Hobfoll, 1989, 2001; Hobfoll et al., 2018), which proposes that resources—such as adaptability (personal) and social support (conditional)—are an important means of protection against current and future stress, and also facilitate the successful management of future stressful situations (Hobfoll, 2001; Dollete and Phillips, 2004). It follows then, that individuals with greater (higher) resources are likely to have more positive psychological wellbeing (Cohen and Willis, 1985; Buzzai et al., 2020): this hypothesis was corroborated in the present research. The results also suggest that effective cognitive, behavioral, and emotional adjustment in situations of novelty and uncertainty (i.e., one’s adaptability, Martin et al., 2012, 2013) is not only a critical element of secondary, pre-16 Students’ psychological wellbeing (Martin et al., 2013), but is also an important factor in the psychological wellbeing of students in non-compulsory education (e.g., Year 12 college education and higher education), and in non-studying adults.

There were some other interesting findings that warrant further discussion. For instance, in Study 2 (using university students) we included a measure psychological distress (Kessler et al., 2002), which focused on experiences of distress over the past 30 days. Here, it was found that adaptability, but not social support, was uniquely associated with this outcome. This can perhaps be explained by the fact that relative to Year 12 college education, university study involves increased independence and personal autonomy; therefore, students at university may have to draw upon personal rather than social resources in order to navigate situations of novelty and uncertainty to avoid stress. Relatedly, university students are more likely to be living away from home; therefore, the availability of family, friends, and significant others (as captured through our measure of social support, Zimet et al., 1988) may be limited and of less significance than at earlier education levels. Additional research with larger sample sizes is needed to further test the generalizability of this finding.

Moreover, in Study 4 [combining the samples from Study 2 (university) and 3 (non-studying adults) to compare the roles of adaptability and social support in relation to life satisfaction and flourishing], we found that while adaptability played a stronger role (than social support) for university students, social support played a stronger role for non-studying adults. This suggests that adaptability may be a more important resource in the context of university than it is for non-studying adults (although it is a unique predictor in both samples). This can perhaps be explained by the fact that many social resources (i.e., support from family, friends, and significant others) are typically less readily availability at university (and perhaps more readily available beyond this context), and therefore, there are stronger demands for self-regulation of one’s own emotions, cognitions, and behaviors, in order to successfully navigate novel environments. Alternatively, it may be that the sheer amount of novelty and change in university (Burnett and Blakemore, 2009) means that this personal resource is more salient for wellbeing outcomes.

One major strength of this paper is that we examined the replicability of the findings reported by Zhou and Lin (2016) by using more heterogeneous samples and a range of psychological wellbeing measures (although it is more accurate to say this was a partial replication). Indeed, it is essential that such novel findings reported in the empirical literature (i.e., that social support was a moderator of the relationship between adaptability and life satisfaction), are tested again considering the recent replication crisis in psychological research (see Open Science Collaboration, 2015; Camerer et al., 2018). We found across three different datasets that there was no evidence of social support being a moderator of psychological wellbeing outcomes. This was also established using a wide range of psychological health and wellbeing outcomes. For instance, there have been calls for studies to incorporate measures of both mental health and mental wellbeing (Hone et al., 2014), and to capture both “hedonic” wellbeing (the affective or “feeling good” dimension, i.e., happiness, life-satisfaction, and positive affect) and “eudaimonic” wellbeing (psychological functioning or “living well” dimension, e.g., social contribution, positive relationships with others, and personal growth/flourishing) (see Schotanus-Dijkstra et al., 2016, for relevant discussion). This study therefore, has responded to such calls, and underscored the importance of adaptability and social support as unique main effects in a wide array of psychological wellbeing and distress outcomes.

### Implications for Practice

The results have shown that adaptability and social support are linked with psychological wellbeing; therefore, one way to positively influence an individual’s psychological functioning may be to focus on their adaptability and/or support networks (these will be considered in turn). To promote one’s adaptability (see Martin et al., 2015), individuals might first be supported to identify situations of change, novelty, and uncertainty, that may require a particular regulatory response. They might then be supported to navigate the situation via adjustments to their emotions (e.g., upward regulation of enjoyment rather than anxiety or frustration), cognitions (e.g., thinking in different ways to find an effective response), and behaviors (e.g., trying new actions that might supersede older ones). Finally, individuals might be supported to recognize the importance of these regulatory responses (and this process), so that new situations can be recognized and managed more effectively in the future. For students, this process might be supported by academics and institutions, who might seek to develop this personal resource and/or adjust the environment and provision to help minimize change, novelty, and uncertainty (Crosling et al., 2009). Equally, non-studying adults may benefit from such endeavors, although engagement with this process may need to be personally instigated and self-directed.

Turning to social support, it is clear that social networks (e.g., friends, family, and/or significant others) may influence an individual’s psychological wellbeing; therefore, any efforts (with students or non-students) designed to promote the availability of social resources are likely to be beneficial. However, this may prove to be more challenging in higher education where, as discussed earlier, such resources are typically less readily available (e.g., students may leave home and old established friendships may cease). However, support in the form of student mentors (see Collings et al., 2014) may be an effective strategy, for discussion and advisement of effective strategies, as well as pastoral care/support that might be available as part of this process. More empirical research is warranted, however, to inform the nature and content of intervention programs that focus on promoting psychological wellbeing via adaptability and/or social support.

### Limitations and Future Directions

In the studies reported here, there are some limitations to acknowledge when interpreting findings. First, as this research utilized quantitative methodology (only), our capacity to “understand” the complexities of apparent trends—and nuances that may be at play for individuals—was somewhat limited. Further research might incorporate qualitative approaches to gain a richer, more insightful understanding of how and when adaptability and social support may operate, for example, in relation to psychological wellbeing for different individuals (see Holliman et al., 2019). Moreover, due to the “self-report” nature of adaptability, social support, and the other measures included in this research, there is the risk of potential inaccurate or biased responding (Podsakoff et al., 2012). Furthermore, by focusing specifically (and solely) on individual-level resources, this research does not adequately capture the wider contexts (i.e., macro factors) in which individuals transition, including systemic challenges at teacher and institution levels (see Green et al., 2015; Vossensteyn et al., 2015); although, by utilizing the COR model and the measure of social support that was chosen, there were efforts to focus on the influence of friends, the family, and the community. Relatedly, it is acknowledged that even with this “individual-level” focus, some potentially important participant details, such as socio-economic status, nationality, and spoken language, were not always recorded. Additionally, as the studies reported here used a concurrent, correlational design, it was not possible to establish cause-effect relations (these can only be conceptually inferred). A measure of behavioral engagement might also have further clarified our understandings regarding the mechanisms by which adaptability (and social support) impacts upon psychological wellbeing outcomes (Martin et al., 2013). Future research might therefore consider using mixed-method, longitudinal designs, which include multiple measures at different time-points, and at different levels, to help establish the pathways of influence and elucidate the nuances that may be at play for individuals in particular contexts. This might also include an examination of possible mediation effects (see Valeri and VanderWeele, 2013) as, in the present work, we focused on independent and moderating effects, to see if the associations reported in prior work (Zhou and Lin, 2016) could be replicated here. Finally, the relatively small sample for Year 12 college students (Study 1) needs to be acknowledged; although it is noteworthy that even with this sample, Study 1 paralleled findings in the other two studies that comprised larger samples. Nonetheless, future studies might investigate the replicability of the findings reported here with different samples and in different contexts, such as in sporting and workplace contexts.

## Conclusion

In this paper, we examined the independent effects of adaptability and social support in relation to psychological wellbeing and, for completeness, we also examined whether there was an interaction effect between adaptability and social support. These findings demonstrate the importance of adaptability and social support in uniquely predicting psychological wellbeing in different sample groups. Moreover, adaptability played a stronger role (relative to social support) in university students, whereas social support played a stronger role for non-studying adults. There was no evidence of an interaction effect between adaptability and social support. Together, these findings hold theoretical and practical implications for educators and researchers who may seek to understand how students (and non-students) manage situations or change, novelty, and uncertainty, and the extent to which personal (adaptability) and conditional (social support) resources are associated with psychological wellbeing outcomes.

## Data Availability Statement

The datasets presented in this article are not readily available because due to the nature of this research, participants of this study did not agree for their data to be shared publicly, so supporting data is not available. Requests to access the datasets should be directed to AH, a.holliman@ucl.ac.uk.

## Ethics Statement

The studies involving human participants were reviewed and approved by the participating University; British Psychological Society. Written informed consent from the participants’ legal guardian/next of kin was not required to participate in this study in accordance with the national legislation and the institutional requirements.

## Author Contributions

All authors listed have made a substantial, direct and intellectual contribution to the work, and approved it for publication.

## Conflict of Interest

The authors declare that the research was conducted in the absence of any commercial or financial relationships that could be construed as a potential conflict of interest.
